# Supplementary material for: Probabilistic controllability approach to metabolic fluxes in normal and cancer tissues
Source: Nat Commun. 2019 Jun 20;10:2725. doi: 10.1038/s41467-019-10616-z (PMC6586789; doi:10.1038/s41467-019-10616-z)
Supplement: Supplementary file 1 — Supplementary Information [file 41467_2019_10616_MOESM1_ESM.pdf]

Supplementary information

Probabilistic controllability approach to metabolic fluxes in normal and cancer tissues

Schwartz et al.

Perturbation analysis for probabilistic controllability

In order to check the stability of controllability results with respect to random alterations in the topologies of the four healthy and cancer networks, we constructed randomised networks by rewiring some proportions of the edges. We used the ‘rewire’ function from the R igraph package, together with the ‘keeping_degseq’ function that preserves the original network’s degree distribution to avoid changing the topological properties of the networks. Supplementary Figures 1, 2 and 3 show comparisons between the PMDS fractions in the original and 10 randomised networks for 1%, 5% and 10% of edges rewired, respectively.

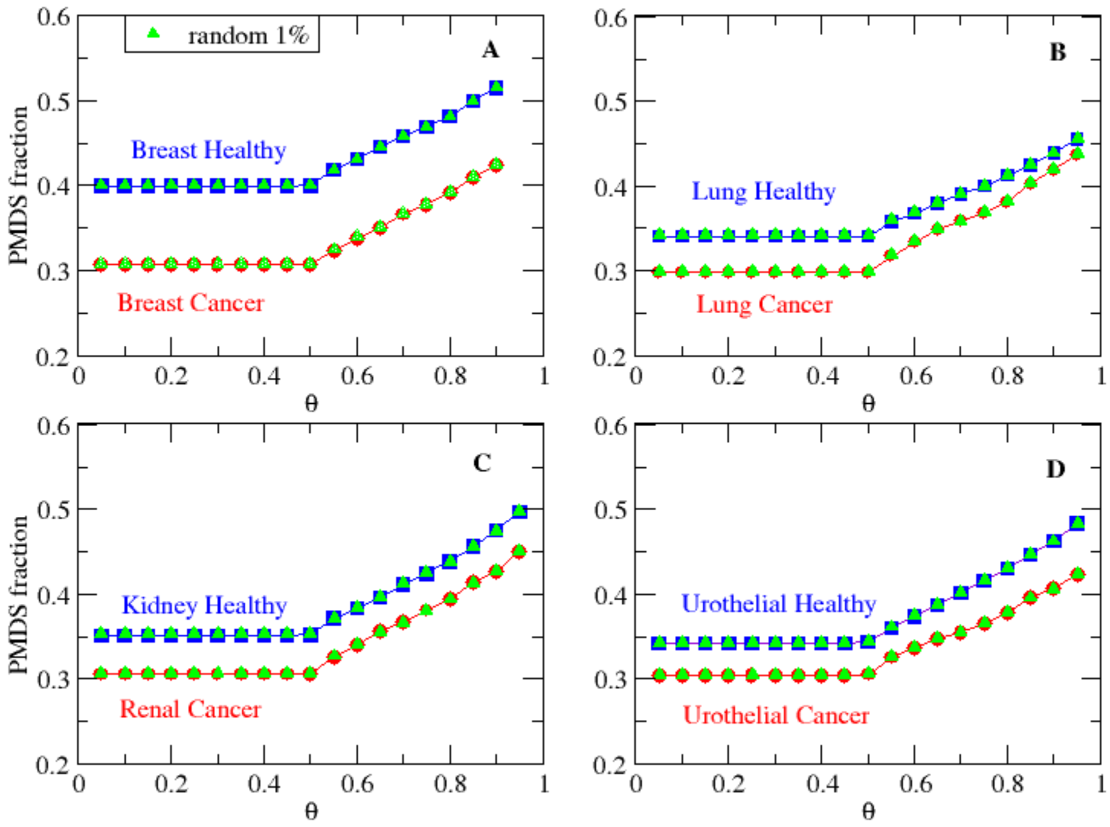

Supplementary Figure 1: Fraction of PMDS in healthy and cancer tissues of breast (A), lung (B), Kidney (C) and Urothelial (D) for different values of the threshold Θ. Red circles: original cancer tissue networks; blue squares: original healthy tissue networks; green triangles: randomised networks with 1% of edges rewired while preserving the original network’s degree distribution. The standard deviation for randomised networks is smaller than ±0.001.

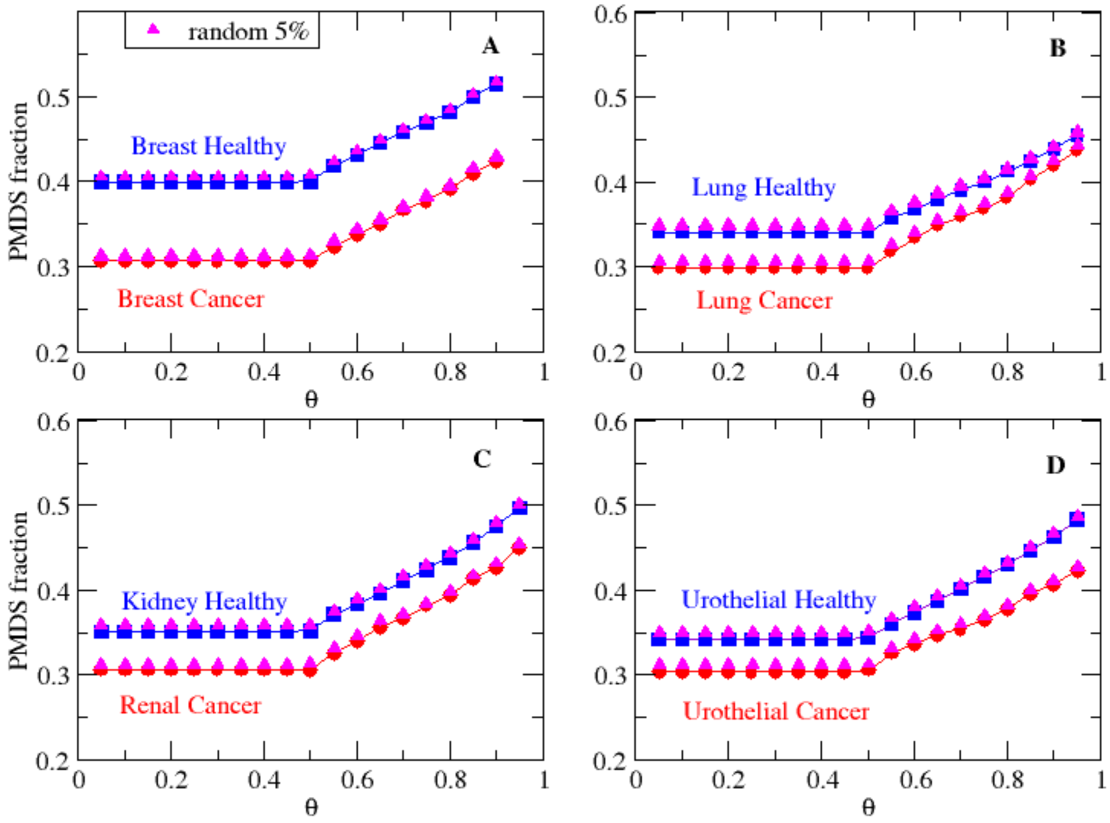

Supplementary Figure 2: Fraction of PMDS in healthy and cancer tissues of breast (A), lung (B), Kidney (C) and Urothelial (D) for different values of the threshold *Θ*. Red circles: original cancer tissue networks; blue squares: original healthy tissue networks; purple triangles: randomised networks with 5% of edges rewired while preserving the original network's degree distribution. The standard deviation for randomised networks is smaller than ±0.003.

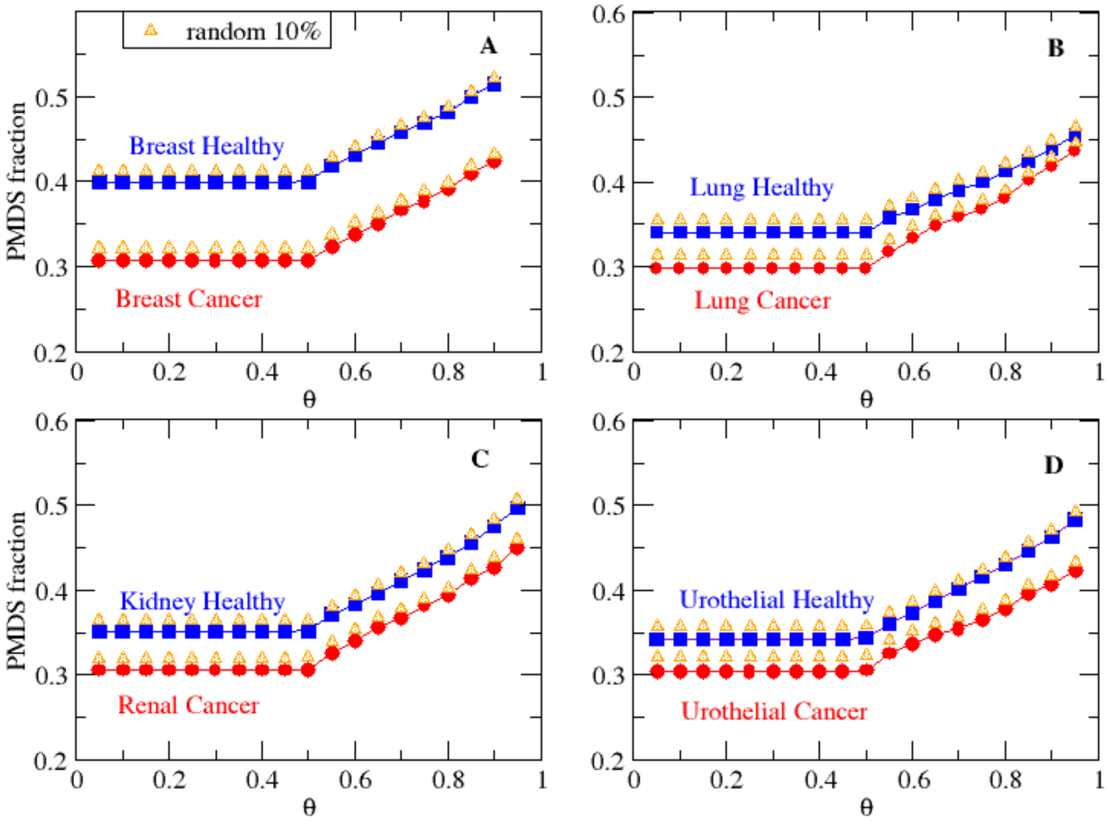

Supplementary Figure 3: Fraction of PMDS in healthy and cancer tissues of breast (A), lung (B), Kidney (C) and Urothelial (D) for different values of the threshold *Θ*. Red circles: original cancer tissue networks; blue squares: original healthy tissue networks; orange triangles: randomised networks with 10% of edges rewired while preserving the original network's degree distribution. The standard deviation for randomised networks is smaller than ±0.004.
